# Supplementary material for: Identification of photoperiod-induced specific miRNAs in the adrenal glands of Sunite sheep (Ovis aries)
Source: Front Vet Sci. 2022 Jul 22;9:888207. doi: 10.3389/fvets.2022.888207 (PMC9354845; doi:10.3389/fvets.2022.888207)
Supplement: Supplementary file 1 [file Data_Sheet_1.ZIP › Supplementary file/miRDeep2 file.zip!/miRDeep2 file/Novel_Structure/LP42A2/Novel_586.pdf]

Provisional ID	:	NC_019463.2_14654
Score total	:	1.7
Score for star read(s)	:	-1.3
Score for read counts	:	0
Score for mfe	:	1.4
Score for randfold	:	1.6
Score for cons. seed	:	
Total read count	:	96
Mature read count	:	96
Loop read count	:	0
Star read count	:	0

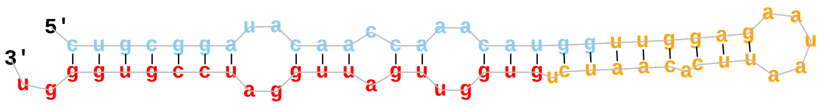

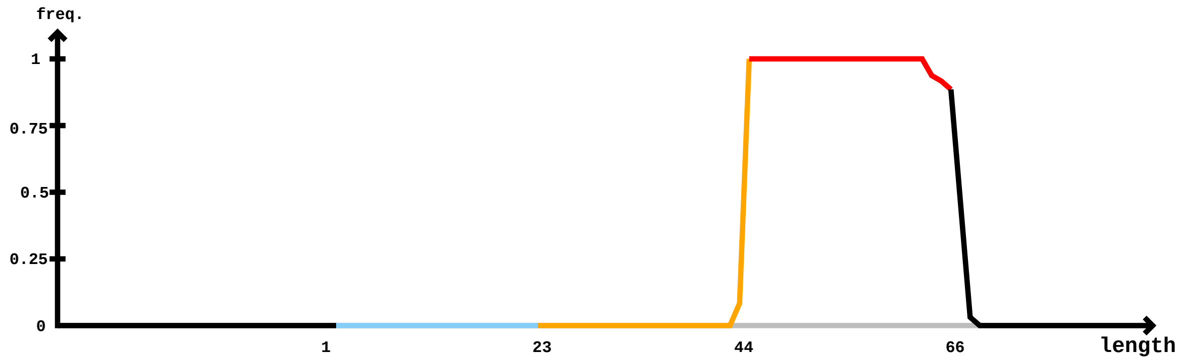


```
                                 Star                                    Mature
5'- agcccuccauaucugugguuucugcaccugcggauacaaccaaacaugguuggagaauaauucacaaucugugguugauuggauccguggguauagaaccugcaaauacaua   -3'    exp
    .........(((.(((((.(((((.(((((((((..(((.((..(((((((((((.....))).))))).)))..)).)))..))))))))).))))).))))).)))....    reads      mm         sample
    ..................................................................ugugguugauuggauccgug......................    3          0          seq
    ..................................................................ugugguugauuggauccgugg.....................    1          0          seq
    ..................................................................ugugguugauuggauccguggg....................    1          0          seq
    ..................................................................ugugguugauuggauccgugggu...................    3          0          seq
    ...................................................................gugguugauuggauccgug......................    3          0          seq
    ...................................................................gugguugauuggauccgugg.....................    1          0          seq
    ...................................................................gugguugauuggauccguggg....................    2          0          seq
    ...................................................................gugguugauuggauccgugggu...................    76         0          seq
    ...................................................................gugguugauuggauccguggua..................    3          0          seq
    ...................................................................gugguugauuggauccgugggau.................    3          0          seq
```
